# Toward the Fabrication of Advanced Nanofiltration Membranes by Controlling Morphologies and Mesochannel Orientations of Hexagonal Lyotropic Liquid Crystals

**DOI:** 10.3390/membranes7030037

**Published:** 2017-07-21

**Authors:** Guang Wang, Christopher J. Garvey, Han Zhao, Kang Huang, Lingxue Kong

**Affiliations:** 1 Institute for Frontier Materials, Deakin University, Locked Bag 20000, Geelong 3220, Australia; guangw@deakin.edu.au; 2 Australian Nuclear Science and Technology Organization, Locked Bag 2001, Kirrawee DC 2232, Australia; cjg@ansto.gov.au; 3 School of Mechanical Engineering, Hefei University of Technology, No. 193 Tunxi Road, Hefei 230009, China; hanzhao@hfut.edu.cn (H.Z.); Hfhuang98@163.com (K.H.)

**Keywords:** nanofiltration (NF), hexagonal lyotropic liquid crystals (hexagonal LLCs), structure retention, reorientation, membranes

## Abstract

Water scarcity has been recognized as one of the major threats to human activity, and, therefore, water purification technologies are increasingly drawing attention worldwide. Nanofiltration (NF) membrane technology has been proven to be an efficient and cost-effective way in terms of the size and continuity of the nanostructure. Using a template based on hexagonal lyotropic liquid crystals (LLCs) and partitioning monomer units within this structure for subsequent photo-polymerisation presents a unique path for the fabrication of NF membranes, potentially producing pores of uniform size, ranging from 1 to 5 nm, and large surface areas. The subsequent orientation of this pore network in a direction normal to a flat polymer film that provides ideal transport properties associated with continuous pores running through the membrane has been achieved by the orientation of hexagonal LLCs through various strategies. This review presents the current progresses on the strategies for structure retention from a hexagonal LLCs template and the up-to-date techniques used for the reorientation of mesochanels for continuity through the whole membrane.

## 1. Introduction

**Figure 1:**
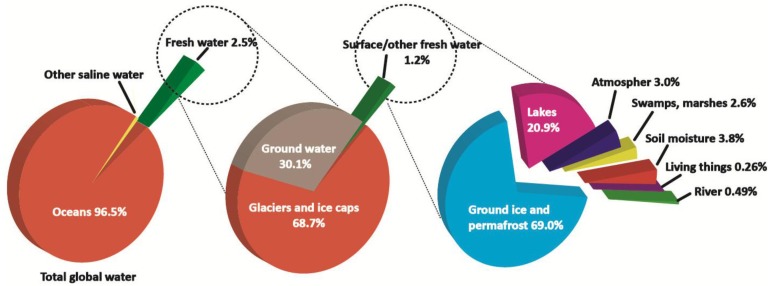
Distribution of water resources on the earth.

Consistent with the data in ‘Water in Crisis’, edited by Gleick [1], the entire body of the world’s water resources is predominantly made up of oceans, with only a relatively small amount of fresh water making up the remaining 2.5%. Of this limited freshwater, only 31.3% is surface and ground water, which can potentially be used by humans, and the remaining 68.7% is found in the form of glaciers and ice caps (Figure 1). Furthermore, many ground and surface water resources have been seriously contaminated due to inadequate sanitation, algal blooms, detergents, fertilizers, pesticides, chemicals, potentially toxic metals, salinity, and high sediment loads. These circumstances have effectively reduced the supply of freshwater for human use [2,3,4,5].

Due to diminishing water resources, desalination has drawn more attention from scientists and governments and has begun playing an important role in some countries’ potable water supplies [6]. However, the high cost involved in desalination, including infrastructure, energy, and the maintenance of facilities cannot be ignored and limits the use of desalination as a solution to potable water shortages. The energy consumption for various seawater desalination methods is shown in Table 1. Although the energy consumption of some desalination methods has almost reached as low as 3 kWh/m^3^, it is still much higher than local fresh water supplies that only use 0.2 kWh/m^3^ or less [7,8]. A state-of-the-art reverse osmosis (RS) system may produce a thousand liters of drinking water by only using ~2.2 kWh of electrical energy; however, this value varies widely with geographical location, height, and distance from the source water [9]. The total cost and increased environmental concerns have limited the widespread adoption of desalination technologies [5]. An economical and environmentally sustainable method of water recycling is therefore essential. The application of membrane technology is widely used for water recycling in terms of cost-effectiveness and environmental friendliness.

The polymer membranes with nanometer size morphologies have great potential to be applied in various areas [13,14,15,16]. Of particular interest here are the separation sieves using organic mesoporous membranes with a uniform pore size in the range of 1–5 nm and a high specific surface area for NF. NF membranes have opened up the possibility of performing highly selective small molecules and water purification based on the molecular size-exclusion of hydrated salt ions [17,18]. A more permeable separation layer with uniform pore distribution will significantly maintain or improve salt rejection while increasing the flux compared to RO. The transport of water molecules through hydrophobic double-walled carbon nanotubes has demonstrated that the fluxes are over three orders of magnitude higher than those predictions using continuum hydrodynamic models [19,20,21]. However, membranes with the necessary anisotropic transport properties due to oriented carbon nanotubes will be difficult and costly to prepare. The high performance of membranes is based on their diameter size (1–5 nm); however, various nanostructured and nanoreactive membranes are encouraged for use in water purification according to diverse pollutants [22,23,24,25,26].

**Figure 2:**
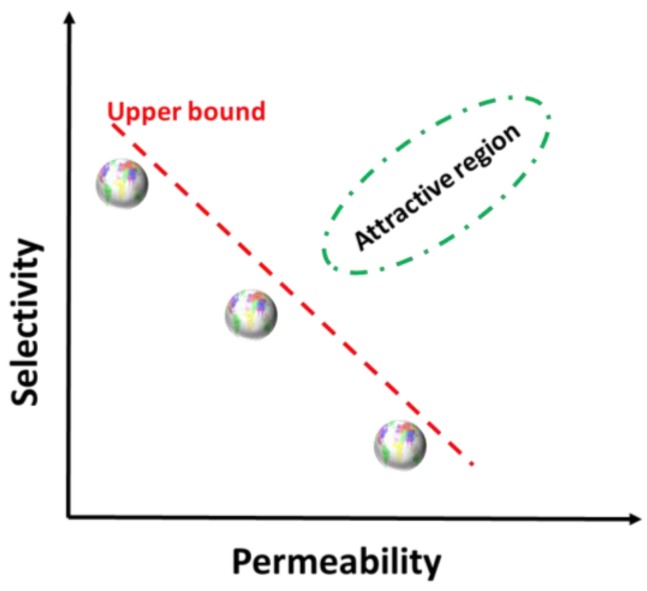
Schematic showing the trade-off between permeability and selectivity of synthetic nanofiltration (NF) membranes.

An ideal membrane for NF would require generating a thin film with physically continuous and vertically aligned nanopores in a narrow size distribution. However, currently developed NF membrane fabrication techniques such as tract etched, particle-assisted wet, and phase inversion precipitation are all suffering from kinetically controlling pores of random size and orientation and are therefore subject to a ubiquitous trade-off between permeability and selectivity, as shown in Figure 2 [27]. Highly permeable membranes lack selectivity and vice versa. The polymer membranes presenting the highest selectivity at a given permeability always lay near or on a line called the upper bound, and this behaviour (the slope), observed in all cases in both dense and porous membranes, absolutely depends on the parameters of the molecules/ion pair [27]. This departs considerably far from the ideal and limits their use. Therefore, for the polymer NF membranes, the ability to precisely control the morphology, size, and anisotropy at a large scale is quite challenging but essential for this application.

**Figure 3:**
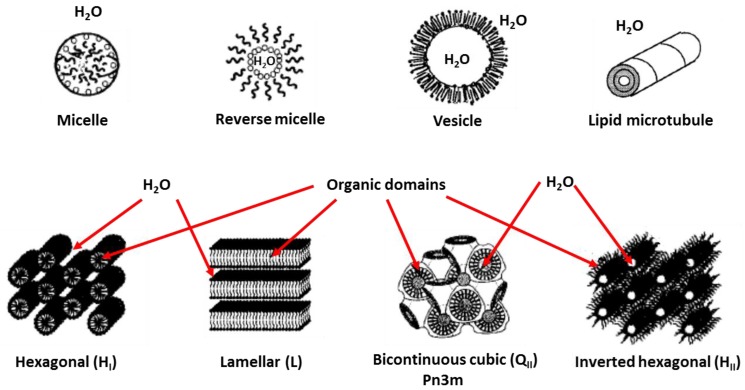
Different types of aggregates formed by surfactants with water [28] (Reprinted with permission from [28]. Copyright (2001) American Chemical Society).

A method for which the hexagonal arrangement of cylinders in LLCs provides a template presents a versatile strategy for the fabrication of NF membranes with uniform and adjustable pore size and large surface area and is promising in terms of overcoming the permeability-selectivity trade-off (attractive region in Figure 2). The template for determining nanostructures is accessible by mixing amphiphiles and a hydrophilic medium like water at a designated ratio under conditions at or close to ambient temperature (Figure 3).

**Figure 4:**
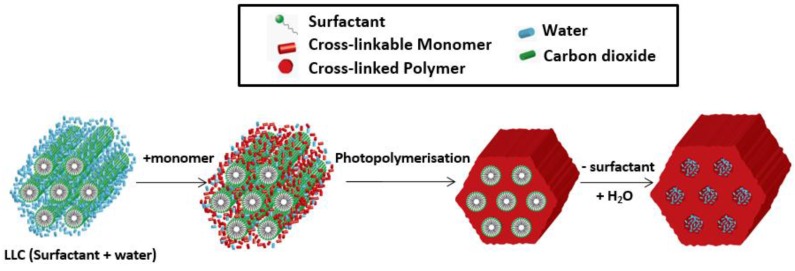
Template method from hexagonal lyotropic liquid crystals (LLCs) for the fabrication of mesoporous membranes. The hexagonal LLCs template is from Dodecyltrimethylammonium bromide (DTAB) and H_2_O [29] (Reprinted with permission from [29]. Copyright (2011) Royal Society of Chemistry).

The hydrophilic and hydrophobic areas within the assembly could be utilized by dissolving them with a polymerizable species, and therefore the nanostructure could be cured after a physicochemical process (Figure 4). The continuity of cylinders (mesochannels) can also be controlled and extended to the bulk materials after reorientation through appropriate strategies. However, the two main difficulties associated with this method are the structure retention during polymerization from monomer to polymer phase and the reorientation of mesochannels in a facile way for high continuity and therefore high flux property. In this review paper, the current progress of the various methods applied for hexagonal LLCs templated-structure retention and the strategies used for reorientation are presented.

## 2. Phase Transition and Retention

### 2.1. Phase Formation

The amphiphilic character of surfactant molecules provides a thermodynamic drive to self-assemble into aggregate structures with well-defined geometry, as shown in Figure 3. The hydrophobic effect is the energetic cost of hydrocarbon/water contact, which is balanced by the interfacial free energy [30]. This drives the assembly after contact with water or other hydrophilic agents, with the heads defining the interfaces of the phase separated aqueous domains and the tails forming hydrophobic regions. Those aggregates consist of simple individual structures such as micelles, vesicles, and highly organized yet fluid condensed assemblies with one-, two-, or three-dimensional nanoscale hierarchical complexes with the increase of the percentage of surfactant in the system. These hierarchical complexes all have a constant lattice ranging from several nanometers to tens of nanometers, which could act as the template candidate for designing novel nanomaterials.

**Figure 5:**
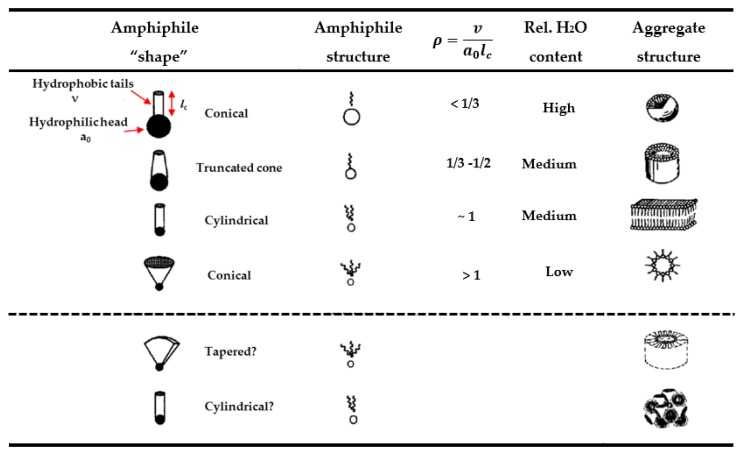
Relationships between the structural dimensions of surfactant molecules and their preferred aggregates according to the Israelachvili Model [31] (Adapted with permission from [31], copyright Elsevier, 1985).

**Figure 6:**
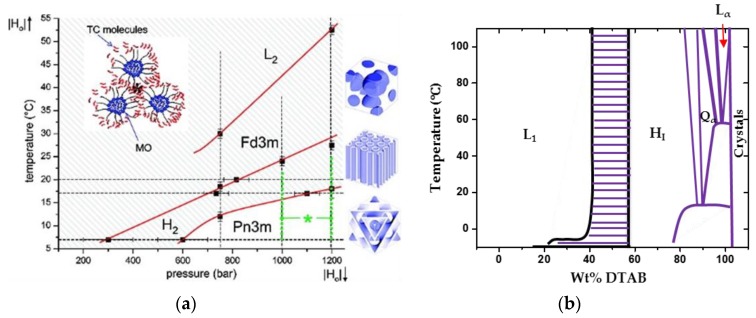
(**a**): Temperature-pressure behaviour of fully hydrated tetradecane-loaded monoolein/water system; (**b**): Temperature-concentration dependent behaviour of LLCs formed of DTAB and water [34,35] (Adapted with permissions from [34,35]. Copyright (2009 and 1995) American Chemical Society).

The self-assembled nanostructures adopting different mesophases highly depend on the molecular shapes and the lipid tail lengths of the investigated surfactants (Figure 5) [32,33,34]. Normally, for a specified surfactant or a mixture of surfactant systems, the assembled nanostructure has temperature, concentration, and pressure dependent behaviours, as shown in Figure 6 [34,35]. Phase transition between different morphologies of aggregates is realizable by adjusting the concentration of the surfactant, the temperature, and the pressure, which can be detected by using a number of techniques, including DSC, NMR, SAXS/SANS, freeze-fracture electron microscopy, and cryo-transmission electron microscopy.

There are two aspects that should be noted about the formation of the LLC morphologies; the bending energy tends to assist surfactants in forming mesophases, which is hindered by the mainly packing frustrations [36,37,38]. The hydrophobic effect that induces segregation into hydrophobic and hydrophilic domains dominates the self-assembly process of the surface-active materials and further forms the ordered mesophases. All of these actions are embodied in the form of curvature on the interface. Actually, the effects of the concentration and the shape of the surfactant molecules on morphologies are all derived from the changes of the curvature on the interface of the aggregates. Two values can be used to judge the curvature at any point on the interface; the mean interfacial curvature, *H*, and the Gaussian curvature, *k* [38,39,40]. *H* is defined as the average of the principal curvatures *C*_1_ and *C*_2_:

(1) $$H=\frac{C_{1}+C_{2}}{2}$$

While *k* is depicted as:

(2) $$k=C_{1}\times C_{2}$$

**Figure 7:**
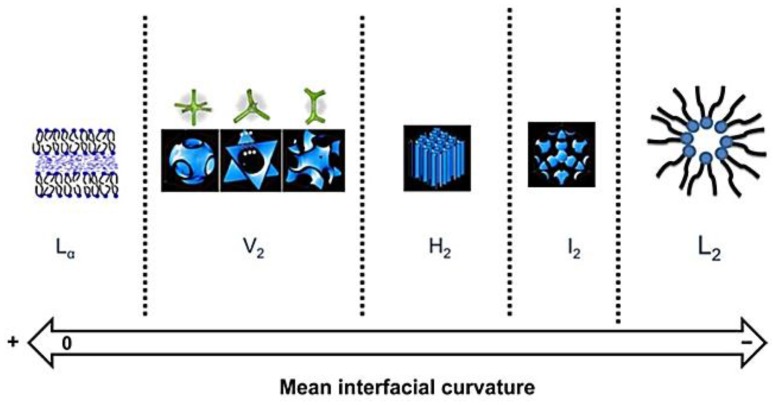
Schematic description for the nanostructure of LLCs depending on the mean interfacial curvature [42]. L_α_: lamellar phase; V_2_: inverted bio-continuous cubic phase; H_2_: inverted hexagonal phase; I_2_: inverted discontinuous cubic phase; L_2_: inverted micellar solution (Reprinted with permission from [42]. Copyright (2013) Elsevier).

Basically, the principal curvature is zero in the lamellar phase, while, in the bicontinuous cubic phase, the principal curvature is nonzero and of the opposite sign: *C*_1_ = −*C*_2_. In the hexagonal phase, one principal curvature is zero and the other one is nonzero [33,41]. The tendency to form inverted mesophases is dictated by increasing the mean negative curvature modulus of the interface, as shown in Figure 7. Normally, the curvature is defined to be negative when the surfaces bend toward the surrounding water and tend to form inverted mesophases; otherwise, it will be positive and prefer to form normal mesophases. The zero state is a flat interface without bend such as lamellar mesophase.

On the other hand, the curvature can also be rationalized on a molecular level. The critical packing parameter (CPP, also called molecular shape factor), *ρ*, is normally used to describe the molecular geometry of the surfactant and the formation of self-assembled structure, which is given by (Figure 5):

(3) $$\rho =V/a_{0}\times l_{c}$$

where *V* is the hydrophobic chain volume, *a*_0_ is the effective hydrophobic/hydrophilic interfacial area, and $l_{c}$ is the hydrophobic chain length.

**Figure 8:**
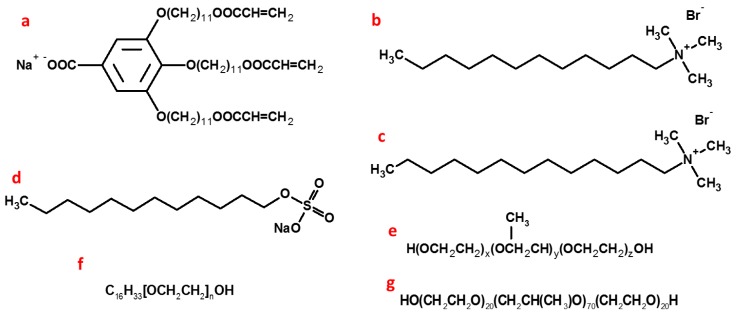
Molecular structure of natural and synthesized amphiphiles mainly used as structure directing agents for hexagonal LLCs. (**a**): NA-GA3C11; (**b**): DTAB; (**c**): Cetyltrimethylammonium bromide (CTAB); (**d**): Sodium dodecyl sulphate (SDS); (**e**): P123; (**f**): Brij56; (**g**): F127.

Actually, the most commonly researched mesophase is the lamellar phase due to the prevalence of this organized lipid structure in living systems. The application of this in the construction of nanostructured organic materials is regarded as one of the most promising research directions. Especially for the hexagonal LLCs that have more highly ordered yet more fluid condensed assemblies than the lamellar phase but less stiffness and a lower elastic property than the cubic phase. Both the normal and inverted hexagonal LLCs have been extensively explored by using synthesized or natural amphiphiles. The amphiphiles, including cationic and anionic surfactants, Gemini surfactant, block copolymers like Pluronic P123 and F127, synthesized NA-GA3C11, Polyethylene glycol hexadecyl ether (Brij56), and some neutral lipidic surfactants like monolinolein and their phase diagrams, have been extensively investigated [43,44,45,46,47,48,49,50]. The molecular structure of those widely used surfactants is displayed in Figure 8.

### 2.2. Phase Transition

Direct templating of hexagonal LLCs has been proven to be a very versatile approach and has demonstrated many advantages with highly ordered networks and narrow pore-size distributions. However, hexagonal LLCs are inherently fluid, and therefore the curvature is sensitive to external factors, although the sensitivity depends strongly on the species and molecular structure of the amphiphiles. For instance, an electric field induced sphere or cubic phase to cylinder transition in a diblock copolymer system and a reversible transition between sphere and hexagonal LLCs under UV light in an azobenzene-containing surfactant system have been reported [51,52,53]. Additionally, long chain alcohol molecules were also found to be able to induce phase transition between hexagonal and layered LLCs in a mixed non-ionic surfactant system by increasing the volume of the hydrophobic moiety of the surfactant system and indirectly leading to the increase of the critical packing parameter, *ρ*, to 1 (Equation (3) and Figure 5). However, the effect absolutely relies on the length of the alcohol molecules compared to the hydrophobic group of surfactant molecules [54]. The interesting tools/methods used to control the phase behaviours present an opportunity to engineer the specific applications via the choice of various structural directing agent and solvents. However, a tough issue in front of us is the phase behaviours that occurring during the shift from a soft template to a robust polymer network during polymerization, as the hexagonal LLCs inherently lack the robustness required for membrane application.

### 2.3. Thermodynamic Behaviours during Polymerization

A facile method to fabricate a mesoporous polymer membrane by using self-assembling LLCs as template is photopolymerization by using diene reactive group. This is advantageous compared with other methods owing to two main reasons:

(1) Self assembling LLCs have a fast initiation rate and are readily controlled through light intensity and photo-initiator concentration [55,56].
(2) LLCs phase behaviours are inherently sensitive to concentration, temperature, and pressure, as mentioned above.

The thermodynamically driven phase separation during polymerization has been regarded as the main reason that prevents polymer morphology from being precisely controlled [57,58]. As the monomer phase is substituted by a polymer phase, the changes in the polarity of the dispersion medium and the partitioning of each compound will occur accordingly. The decrease of entropy and the reduction in enthalpy mainly originate from the loss of freedom of the growing polymer chain that accompanies polymerization [59]. Research has demonstrated that, while keeping the double bond concentration constant, the hexagonal LLCs exhibited the slowest polymerization rate compared to the lamellar and even to the cubic phase [60]. Additioanlly, photoinitiation efficiency is also a function of viscosity, polarity, and the diffusional constraints inherent in LLCs phases [61]. The extensive research on the influence factors, including monomer polarity and its location, photoinitiator polarity and its mobility, and the initiation rate and reaction speed that can affect the polymerization kinetics and therefore the structure retention in an LLCs system, have been reported by Guymon’s group [60,61,62,63,64,65]. In their research, the polarity and mobility of the monomer and the photoinitiator played an important role in influencing the polymerization kinetics. The mobile and hydrophilic initiators have shown an independent initiation rate in LLCs systems of various compositions and morphologies. The initiation rate of the immobile and hydrophobic ones appears highly dependent on the order and composition of the LLCs. The initiation efficiency with relatively bulky, hydrophobic initiators increases significantly in more ordered systems, while it decreases slightly for less hydrophobic initiators in a more ordered phase. When partitioning the polar monomers into a continuous phase in normal LLCs, they will present an increasing polymerization rate in more ordered LLCs. However, the non-polar monomers exhibited an opposite trend due to the segregation effect in the discontinuous area. Additionally, the conversion rate from monomer to polymer significantly increased during polymerization under a non-oxygen environment [66]. The experimental and theoretical aspects of thermodynamically controlled phase behaviours and polymerization kinetics have to be linked to reflect a delicate balance for structure retention in the final product materials.

Apart from polymerization, the treatments for the final membrane product that we followed, including surfactant removal and dehydration, are all critical to retaining the morphology of the precisely controlled nanostructure.

### 2.4. Phase Retention

The disruption of the inner structure during polymerization is the main obstacle for the fabrication of organic mesoporous membranes. To resolve this problem, some considerations could be taken to retain the desired phase:

- The location of diene groups conjugated with the acyl chain carbonyl should not interfere with the biocompatibility of the interface and have less effect on the curvature;
- The entropy loss and enthalpy reduction should be compensated by using the monomers with a high number of reactive entities so that the monomers can be polymerized with the lowest conversion rate;
- The molecular structure of the surfactant or surfactant mixtures or any other molecules dissolved in the system is also important to predict the preference of the packing pattern [37,67];
- A more thermodynamically stable template with long rearrangement times should be developed; and
- Some small multifunctional monomers with high mobility that form cross-linked networks easily could be used to enforce the instant interface curvature during polymerization.

A large number of efforts have been made to conquer the phase transition/separation of hexagonal LLCs to increase the retention rate of a nanostructure during polymerization and the following processes such as surfactant removal and dehydration. The developed methods based on the considerations above are summarized as follows.

#### 2.4.1. Application of Cosurfactant

Cosurfactants play an important role in directing the formation of a mesophase, stabilizing the LLCs at the required mesophase and controlling the alignment process by acting as structure directing agents, charge transfer complexation agents, hydrophobic swelling agents, or stabilizers [45,50,68,69]. The main mechanism is that cosurfactants will partition at the interface between the hydrophobic and hydrophilic domain and reduce the repulsive interaction of the adjacent head group, by which they can control the curvature and stabilize the current phase. For some alcohols with medium or long chains, they could diffuse into the hydrophobic domain and transfer the current phase to another phase because of the swelling effect. Based on these mechanisms, co-surfactants could also be an effective tool to make the hexagonal phase stable or realize the phase transition [51,53]. Additionally, the pore size of the nanochannels could be controlled by changing the mass ratio of the surfactant/co-surfactant as well [70].

#### 2.4.2. Surfactant Analogues

**Figure 9:**
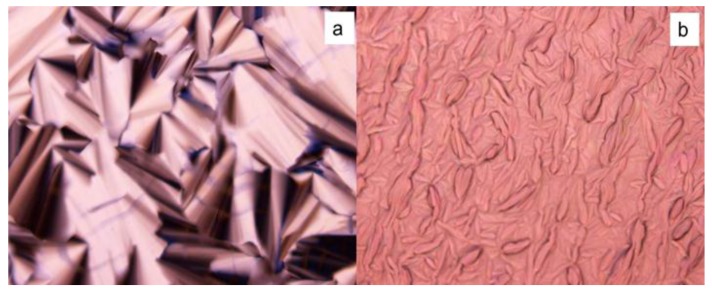
Polarized light microscope images before (**a**) and after (**b**) photo-polymerization.

**Figure 10:**
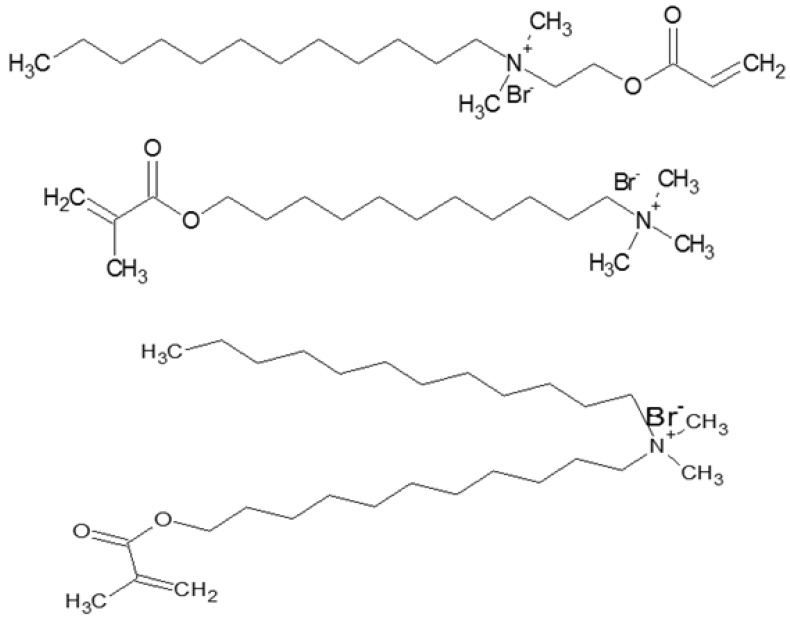
Synthesized surfactant analogues of DTAB from previous research.

Thermodynamically driven phase separation within surfactant mesophase often prevents polymer morphology from being precisely controlled after polymerization, which further leads to the changes in the physicochemical properties of fabricated materials that highly depend on nanostructures [71,72]. Previous results demonstrated that the birefringence decreased and the fan-like optical texture was less defined after polymerization (Figure 9). Meanwhile, the scattering intensity decreased and the position of scattering peaks changed due to the less ordered nanostructure. Research also showed that the phase separation during polymerization is the result of a decrease of entropy originating from the loss of the conformational freedom of the growing polymer chain in the LLCs template, combined with a reduction of enthalpy due to the polymers staying solvated within the amphiphiles [59,73]. Therefore, the analogues of the surfactant were synthesized by grafting the diene group into the heads or tails of surfactants to improve the compatibility between the monomer and surfactants and to counterbalance the loss of entropy during photopolymerization, by which the retention rate of the hexagonal structure was significantly improved after polymerization. Incorporating a low level of such synthesized reactive (polymerizable) surfactants and some highly active small molecules used as crosslinkers was found to facilitate the retention of the order of the LLCs after polymerization [59]. Additionally, the mixture of different polymerizable surfactants was also confirmed to be able to control the hexagonal phase curvature. The molecular structure of the polymerizable surfactants synthesized by using DTAB molecules is displayed in Figure 10 [59,74]. Alternatively, the synthesized surfactant molecules with polymerizable hydrophobic areas and special molecular shapes that prefer to form the stable hexagonal packing based on the Israelachvili Model are also good choices to increase the retention rate (Figure 8a) [28,75].

Apart from the efforts from a chemistry perspective, physically reinforcing the nanostructure by introducing a robust silica network to retain the nanostructure was recently proven to be an effective route [76].

#### 2.4.3. Special Drying Process

The fabrication of mesoporous membranes will need several processes after polymerization. This will give another challenge as to whether the polymerized nanostructure could be further retained during processes such as surfactant removal and the dehydration process. The water will inevitably diffuse into the template even in a polymerized system. In this case, it is generally believed that a higher cross-link density by incorporating some small multifunctional monomers or the monomers with a high number of reactive groups would significantly decrease the effect of hydration on the polymer system. On the other hand, research has shown that maintaining low surface tension during the dehydration process was the key to retaining the original structure of the materials. The critical point carbon dioxide (CO_2_) drying method with lower or even zero surface tension proved to be an efficient way to enhance the internal filamentous structure of biofilm and porous materials, including organic and inorganic materials [29,77,78]. The fundamental is that the surface tension remains constant during the whole drying process, including substituting the water with liquid CO_2_ and followed by drying the membrane at the CO_2_ critical point at which the densities of the liquid and gaseous phases are identical.

## 3. Reorientation

The retention of the hexagonal phase is the prerequisite for the fabrication of mesoporous membranes with porosity. The membranes with controllable nanostructure dimensionality such as pore size and wall thickness but favourable orientation are very important materials for further applications in the treatment of surface water, groundwater, and industrial wastewater in which different contaminants such as toxic metals, organic and inorganic compounds, bacteria, and viruses could be found. The dimensional elements of the hexagonal packing include pore size, water and head group thickness, and the average area available to each head group and could be calculated by using the volume fraction of amphiphiles and water molecules from SAXS/SANS measurement [79,80]. To some extent, the pore size could be also adjustable by using some organic molecules [70]. However, the inherent preference of the isotropic distribution of the mesochannels within the template severely limits the filtration efficiency even if the nanostructure is precisely controlled during the whole process. In addition, the mechanical property will be significantly improved for a monolith after aligning the cylinders through the film. Therefore, the continuity of nanopores through the whole film by reorientating the mesochannels will be another key obstacle for the actual application.

Polymer membranes with well-defined pores in the range of 1–5 nm have the potential to selectively distinguish and transport certain molecules or ions based on their shape, size, and chemical properties. Several methods have been developed to align the hexagonal mesochannels, with the long range being parallel or perpendicular to the substrate according to the actual requests. However, in contrast to the pores with their long axis parallel to the substrate, the materials with perpendicularly aligned channels in respect to the film surface are obviously more versatile for optoelectronic devices, separators with high flux, ultra-high-density recording media, dand novel controlled drug release systems [15,81,82,83]. The strategies developed to align mesochannels templated from hexagonal LLCs are comprehensively discussed, as follows.

### 3.1. External Field

**Figure 11:**
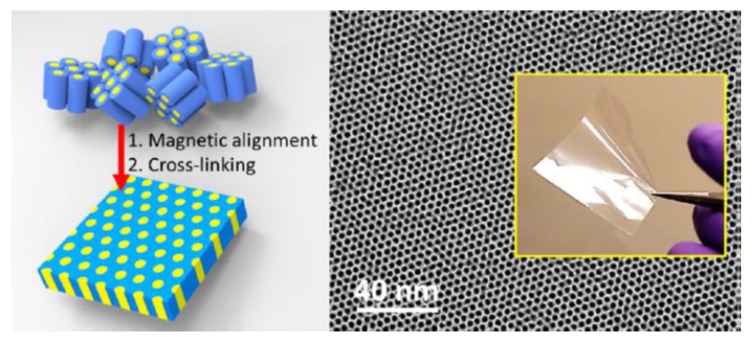
Prepared mesoporous membrane templated from hexagonal LLC and its TEM images after reorientation under a strong magnetic field [47] (Reprinted with permission from [47]. Copyright (2014) American Chemistry Society).

The application of commonly used external fields, including magnetic fields, electrical fields, and shear force, has shown a powerful ability to administrate the orientation of mesochannels. Magnetically induced anisotropy alignment has been achieved during the last few years by mainly using the high magnetic field of the NMR spectrometer permanent magnet [84,85]. Deuterium NMR spectroscopy can be used to characterize the aggregate structure and to quantify the degree of alignment. The overall diamagnetic susceptibility of the aggregates is the key parameter that administrates the orientation with respect to the field direction. The overall orientation of the mesochannels could be easily controlled by incorporating the molecules with large positive/negative diamagnetic susceptibility and therefore adjusting the overall diamagnetic susceptibility of the aggregates [84]. The field strength, on the other hand, is another key parameter to decide the degree of alignment. Due to the negative diamagnetic susceptibility of the alkyl chains in many surfactants and the low magnetic susceptibility of all surfactant molecules, the alignment normally requires a very high magnetic field (>3 Tesla) [86]. Very recently, transparent membranes with vertically aligned 1 nm pores have been reported after reorientation under a magnetic field with a strength higher than 3 T (Figure 11) [47,87]. Additionally, the research found that introducing ferromagnetic nanoparticles into the hexagonal phase can make the nanostructures more easily aligned in a low magnetic field [50]. However, the necessary condition is that the mesophase periodicity has to be larger than the diameter of the nanoparticles. The aggregation and phase separation of nanoparticles inside the system always confine the application of the magnetic field.

The electrical field is another promising tool to align the mesochannels in films. It was initially applied for the alignment of the lamellar mesophase [88]. However, for non-lamellar mesophases, research has shown that the electric field can induce the phase transition between the hexagonal and cubic phase by the interaction between the electric field and the materials via different mechanisms [51,53,89]. For dielectric materials, the dipole (polarization) effect can be used to orient liquid crystals, for which a high strength is normally needed (*E* > 10^5^ V/m) [90], while, in a system with charges, the electrokinetic effect will be sensitive to the applied strength and therefore the required strength is less [91]. However, some inherent disadvantages like electrode contact issues and electrical breakdown concerns always limit the actual application, and therefore using anelectric field for reorienting hexagonal LLCs has not been reported on extensively.

**Figure 12:**
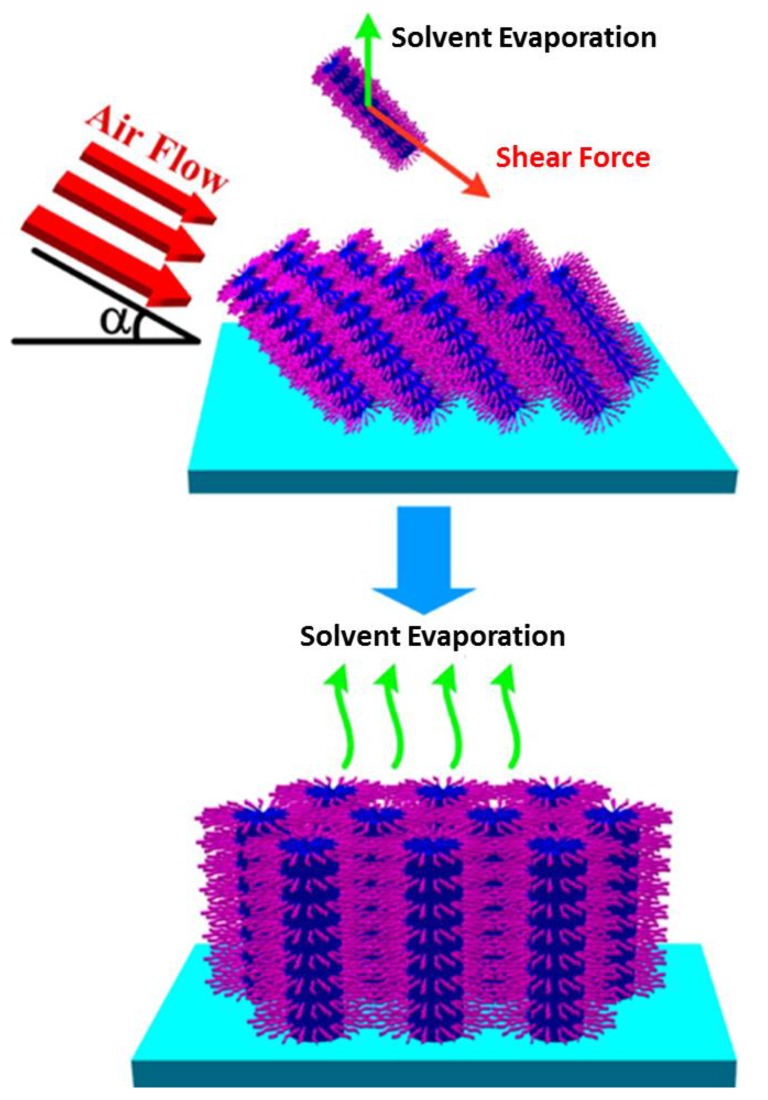
Illustration of the process for vertically aligned mesochannels by using shear force [46] (Reprinted with permission from [46]. Copyright (2012) American Chemical Society).

Shear force, as another important external field to induce alignment, was first demonstrated by Hillhouse [92]. Normally the mesochannels in the hexagonal phase, with their direction aligned along the flow direction (air flow or water flow), and the induced shear force can guide the reorientation of tubular domains according to the flow rate, flow directions, and the ambient conditions [46,93,94]. Sometimes, the other external forces can be used together with the shear force to improve the reorientation effect (Figure 12).

### 3.2. Confinement in a Small Space

**Figure 13:**
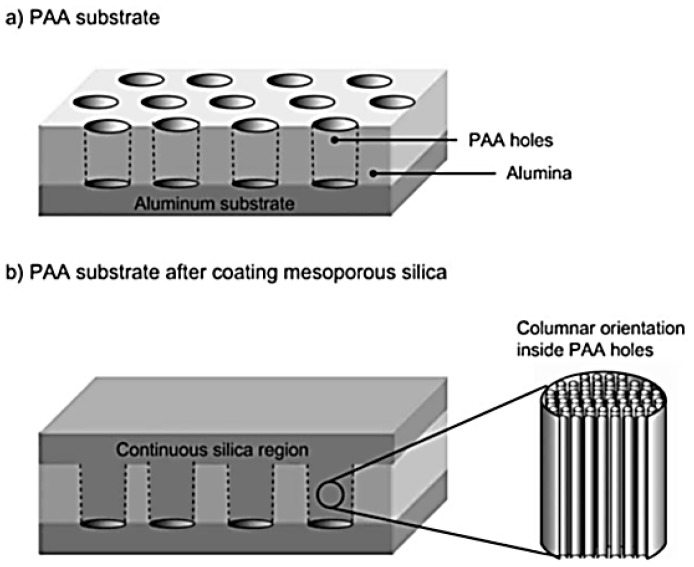
Diagram showing the formation of vertically aligned nanoholes by confining the LLC precursor into the nanoholes on polyacrylic acid (PAA) substrate [96] (Reprinted with permission from [96]. Copyright (2009) WILEY-VCH Verlag Gmbh & Co.).

It is possible to align the mesochannels when the hexagonal LLCs are formed in a small space as the structure of crystals with long axes will form along with a specified direction. Research has shown that, when CTAB was introduced into the matrices of polyacrylic acid (PAA) channels as a structural directing agent, the mesochannels of hexagonal aggregates were mostly parallel to the PAA channels under suitable conditions (Figure 13) [95,96]. In addition, some researchers also used this micro- or nano-space in situ with the external field or some other effects to align the mesochannels [97,98]. However, this method is difficult to apply in membrane fabrication.

### 3.3. Other Methods

Apart from the ways mentioned above, some other methods were also developed to align the mesochannels in hexagonal LLCs and have shown potential value. The most commonly used method was the modification of the substrate surface. Some scientists reported that the mesochannels were prepared and aligned on mica, graphite, silica, quartz glass, and polymer films, which acted as substrates, by using a substrate-molecule interaction that definitely regulates the orientation of mesochannels [99,100,101]. Interestingly, a versatile strategy to achieve the perpendicular alignment by the π-π interaction between organic template molecules and 2-dimensional π plane graphite or silicon wafer surfaces with different surface energies was reported [68]. Meanwhile, the application of the electrochemically assisted alignment of the mesochannels in silica films was explored, in which various conducting supports and even electronic paper were explored to conduct the alignment of the mesochannels [102,103]. In addition, a series of novel Gemini surfactants, which have a high charge density and two polar head groups, were also successfully used to prepare the mesoporous membranes with mesochannels normal to the membrane surface [45]. Most recently, a simple method called the stöber-solution growth approach was explored to prepare highly ordered perpendicular mesochannels transformed from uniform mesoporous nanospheres after a series of interactions between components in stöber-solution under suitable conditions [44].

## 4. Future Directions and Conclusions

### 4.1. Future Directions

The introduction of the silica network has been found to reinforce the hexagonal LLCs template and significantly boost the structure retention rate in combination with the CO_2_ critical point drying method from our previous work [29,76]. Researchers have been trying to control over the phase curvature chemically or physically to increase the retention rate of nanostructures. However, an important issue overlooked by the researchers but that should be emphasised is the distribution of the monomer(s) within the hexagonal LLCs template, which could also be a critical mechanism that can control the phase retention and its filtration efficiency. Research has demonstrated the preferred distribution of the water molecules and the solubilised species within the lamellar phase [104,105]. Meanwhile, the physical robustness of the polymerized materials highly depends on the thickness of hydrophilic area, where the thickness of pore wall comes after polymerization. Undoubtedly, the thicker water channel and the evenly distributed monomer(s) are preferable for the robustness of membranes. However, all this detailed information on the thickness of the hydrophilic area and the distribution of the water/monomer(s) within the hexagonal LLCs has not been explored.

A suitable thin yet mechanically robust film will significantly improve the permeability of NF processes. The inherent viscoelasticity of the hexagonal LLCs makes it troublesome to prepare a membrane template precursor with an exact thickness. The robustness, on the other hand, sets a lower threshold on the thickness of the film. Fortunately, a reversible phase transition as a function of temperature could be realized by incorporating small molecules into the template without significantly affecting the structure. Those small molecules enable the further process of the samples in a flow regime at as low as about 45 °C, depending on various systems, via molecular design, thereby enabling the thickness to be controlled after cooling to room temperature. The introduction of small molecules with a high number of reactive groups also helps to increase the cross-linking density and further benefits the mechanical property. Additionally, alcoholic molecules such as methanol or ethanol are also good options to disperse the samples under a flow state; after that, the desired membrane thickness could be obtained by evaporating the alcoholic molecules. However, the evaporation process should be carefully maneuvered to maintain the water contents in the template.

Further, to get a monolithic membrane template for the fabrication of mesoporous membrane and improve its mechanical properties, the facile and scalable methods for the reorientation of mesochanels are essential to put the fabrication of mesoporous membrane templated from hexagonal LLCs into actual practice. The most commonly used method for reorientation is mainly the strong magnetic field (>3 Tesla) [87]. However, a more promising method probably should be the electric field, which presents a similar reaction mechanism, as an electric field is easily accessible in the laboratory and in actual practice. Although it has some disadvantages such as contact issues and breakdown concerns compared to the magnetic field, those concerns can be overcome by appropriate design. More importantly, the electric field could provide an excellent compatibility with thin film geometries and is inherently scalable from the lab to actual application.

Finally, due to an increasing emphasis on environmental concerns, the renewable/sustainable materials used as the structure-directing agent or the reactive monomer will gain more attention. Recent research for the NF membranes polymerized by using plant-derive fatty acid molecules templated into highly ordered columnar mesophase opened up a promising route for the sustainable development of NF membranes [106]. Actually, the amphiphile, rich in living systems such as plants or animals, is environmentally friendly and could be considered a good potential candidate for membrane materials.

### 4.2. Conclusions

The template method from hexagonal LLCs provides a facile and scalable path for the fabrication of mesoporous membranes with a pore size in the range of 1–5 nm and large surface areas. The thermodynamically controlled phase behaviours and the separation from the decrease of entropy and the reduction in enthalpy during polymerization have been proven to be the main reason for structure distortion. Several considerations and strategies were given to compensate the thermodynamic changes during polymerization, and further processes followed for structure retention. The retention rate has been significantly improved for mesoporous membrane fabrication, accordingly.

The other key part discussed in the paper is the alignment of mesochannels for continuity through the whole film. Various techniques have been developed for the reorientation of mesochannels like external fields, electrochemical methods, special interaction, or novel designed structure-directing agents. However, those methods still need to be further developed to improve their effectiveness and scalability, although some of them have been applied to achieve a good transparent membrane product.

## Figures and Tables

**Table 1:** Energy consumption of various desalination methods [10,11,12].

Desalination Method	MSF	MED	MVC	RO
Electrical energy (kWh/m^3^)	4–6	1.5–2.5	7–12	3–3.5
Thermal energy (kWh/m^3^)	50–100	60–100	None	None
Total equivalent electrical energy (kWh/m^3^)	13.5–25.5	6.5–11	7–12	3–5.5

MSF: Multi-stage Flash; MED: Multi-Effect Distillation; MVC: Mechanical Vapour Compression; RO: Reverse Osmosis.
